# The effect of the pro-inflammatory cytokine tumor necrosis factor-alpha on human joint capsule myofibroblasts

**DOI:** 10.1186/ar2902

**Published:** 2010-01-08

**Authors:** Stefan G Mattyasovszky, Alexander Hofmann, Christoph Brochhausen, Ulrike Ritz, Sebastian Kuhn, Jochen Wollstädter, Hendrik Schulze-Koops, Lars P Müller, Bernhard Watzer, Pol M Rommens

**Affiliations:** 1 Department of Trauma and Orthopaedic Surgery, Johannes Gutenberg University School of Medicine, Langenbeckstr. 1, 55101 Mainz, Germany; 2 Institute of Pathology, Johannes Gutenberg University School of Medicine, Langenbeckstr. 1, 55101 Mainz, Germany; 3 Division of Rheumatology, Medizinische Poliklinik, Ludwig Maximilians University, Pettenkoferstr. 8a, 80336 Munich, Germany; 4 Eicosanoid and Mass Spectrometry Laboratory, Mother-Child Medical Center, Baldingerstrasse, 35043 Marburg, Germany

## Abstract

**Introduction:**

Previous studies have shown that the number of myoblastically differentiated fibroblasts known as myofibroblasts (MFs) is significantly increased in stiff joint capsules, indicating their crucial role in the pathogenesis of post-traumatic joint stiffness. Although the mode of MFs' function has been well defined for different diseases associated with tissue fibrosis, the underlying mechanisms of their regulation in the pathogenesis of post-traumatic joint capsule contracture are largely unknown.

**Methods:**

In this study, we examined the impact of the pro-inflammatory cytokine tumor necrosis factor-alpha (TNF-α) on cellular functions of human joint capsule MFs. MFs were challenged with different concentrations of TNF-α with or without both its specifically inactivating antibody infliximab (IFX) and cyclooxygenase-2 (COX2) inhibitor diclofenac. Cell proliferation, gene expression of both *alpha-smooth muscle actin (α-SMA) *and *collagen type I*, the synthesis of prostaglandin derivates E_2_, F_1A_, and F_2A_, as well as the ability to contract the extracellular matrix were assayed in monolayers and in a three-dimensional collagen gel contraction model. The α-SMA and COX2 protein expressions were evaluated by immunofluorescence staining and Western blot analysis.

**Results:**

The results indicate that TNF-α promotes cell viability and proliferation of MFs, but significantly inhibits the contraction of the extracellular matrix in a dose-dependent manner. This effect was associated with downregulation of *α-SMA *and *collagen type I *by TNF-α application. Furthermore, we found a significant time-dependent upregulation of prostaglandin E_2 _synthesis upon TNF-α treatment. The effect of TNF-α on COX2-positive MFs could be specifically prevented by IFX and partially reduced by the COX2 inhibitor diclofenac.

**Conclusions:**

Our results provide evidence that TNF-α specifically modulates the function of MFs through regulation of prostaglandin E_2 _synthesis and therefore may play a crucial role in the pathogenesis of joint capsule contractures.

## Introduction

Post-traumatic joint stiffness is a common complication that occurs primarily after injuries of the upper extremities involving articular structures [1,2]. In the majority of cases, loss of function after trauma is due to adhesions and contractions as well as to scar formation within capsulo-ligamentous structures. Upon injury, fibroblasts of the surrounding tissue become activated, start to proliferate, and undergo a phenotypic differentiation into contractile myofibroblasts (MFs) [3]. Differentiated MFs are characterized by the expression of alpha-smooth muscle actin (α-SMA), a protein that is associated with the contractile phenotype of this cell type [4-6], as well as the synthesis of proteins over the course of the healing process [5-8]. Although the underlying mechanisms of joint capsule contracture are still poorly understood on the cellular level, the activation and differentiation of MFs seem to be controlled by a complex tissue-specific network of growth factors and cytokines [3,9]. Mechanical stress, ED-A (extra domain A) fibronectin, and transforming growth factor-beta 1 (TGF-β1) are potent inducers of α-SMA expression and thus are considered to be pro-fibrotic factors [3,5,6,10,11]. The complex interaction of different growth factors, cytokines, and extracellular matrix (ECM) may create an environment with an abnormal cytokine profile, which triggers the excessive formation of MFs followed by high matrix turnover. In this context, numerous fibroconnective disorders [8,10] such as Dupuytren contracture [12,13], carpal tunnel syndrome [14], and frozen shoulder [15] have been associated with the appearance of MFs.

Until now, it has not been clear whether a specific inhibition of certain cytokines would be beneficial for prevention of unrestricted MF activation. The pro-inflammatory cytokine tumor necrosis factor-alpha (TNF-α) has aroused our interest as a potential target molecule since the results of recent studies have demonstrated its antagonistic activity against the pro-fibrotic factor TGF-β1 [16-18]. These findings, however, still need further confirmation as the effect of TNF-α may be site- as well as organ-specific. TNF-α may exert direct cellular effects on TGF-β1 expression, as shown by Sullivan and colleagues [19] for lung fibroblasts. Moreover, TNF-α is capable of regulating the activity of cardiac fibroblasts by decreasing collagen synthesis and increasing matrix metalloproteinase activity [20]. However, the role of this pleiotropic cytokine has not yet been defined in the pathogenesis of post-traumatic joint contracture. Based on the current data, we hypothesized that TNF-α is likely to modulate the proliferation, differentiation, and function of human joint capsule MFs and therefore may unveil new therapeutic approaches for the prevention and treatment of post-traumatic joint contracture. Here, we describe the effect of TNF-α and its specific inhibitor infliximab (IFX) on human MFs under controlled *in vitro *conditions. We also report that the positive regulation of prostaglandin E_2 _(PGE_2_) by TNF-α may play an important role in the regulation of human joint capsule MF function.

## Materials and methods

Human hip joint capsules were taken from 12 adult patients (10 women and 2 men) with a mean age of 73 years (range 58 to 96) undergoing orthopedic surgery. The original injuries were either displaced femoral neck fractures (n = 6) or advanced osteoarthritis (n = 6) treated with hemi-hip or total hip replacement. Physical examination in terms of the range of motion (ROM) of the hip joints in patients with fractures was not possible. However, based on the medical history, there were no indications about restricted ROM before the injury. The six patients operated on for osteoarthritis revealed a mean ROM of the hip joint as follows: extension/flexion 0°-0°-108°, external/internal rotation 25°-0°-20°, and abduction/adduction 50°-0°-20°. The patients included neither were operated on before nor suffered from rheumatic diseases or any conditions known to affect wound healing.

For immunohistochemical comparison, contracted elbow joint capsules were taken from four patients (3 women and 1 man; mean age of 61 years, range 56 to 65) undergoing elbow surgery for post-traumatic stiffness. The original injuries of the elbow patients were comminuted distal humeral fractures treated primarily with open reduction and internal fixation (ORIF) with plates. All of the patients operated on had stiff elbows with severely limited ROM.

All of the functional experiments were performed with cells isolated from hip joint capsules at least in triplicate using triplicate or quadruplicate samples, whereas the number of measurements in probands varied for technical reasons. The joint capsules used for the study were considered to be surgical waste and otherwise would have been discarded by the hospital. All experiments were approved by the local ethics committee of Rheinland Pfalz (RLP 837.109.05 [4767]), and written informed consent was obtained from every participating patient.

### Cell isolation and culture of human myofibroblasts

All of the cultures and functional experiments were performed with cells isolated from hip joint capsules. The joint capsules were processed within 6 hours after excision. The inner layer of the capsule, the synovial membrane, which was loosely attached to the external fibrous capsule, was carefully dissected from the fibrous tissue. For all of the experiments, the outer layer of the joint capsule with the fibrous tissue was used. The samples were rinsed in phosphate-buffered saline (PBS) (Dulbecco's PBS; Gibco Invitrogen Corporation, Karlsruhe, Germany) to remove blood and fat residues and were gradually digested in a water bath at 37°C with a mixture of type IV collagenase (1 mg/mL; Sigma-Aldrich Chemie GmbH, Steinheim, Germany), trypsin (2.5 mg/mL; Sigma-Aldrich Chemie GmbH), and DNase I (deoxyribonuclease I) (2 mg/mL; Applichem GmbH, Darmstadt, Germany). The specimens were filtered through a cell strainer (100-μm mesh; BD Biosciences, Heidelberg, Germany) after 45 and 90 minutes of incubation to obtain a single-cell suspension. The cell supernatant was washed in serum-free Dulbecco's modified Eagle's medium (DMEM) (Biochrom AG, Berlin, Germany) supplemented with 10,000 U/mL penicillin G sodium and 10,000 μg/mL streptomycin sulfate (Gibco Invitrogen Corporation, Karlsruhe, Germany) and finally centrifuged at 1.4 × 10^3 ^rpm for 5 minutes at 4°C. The cell pellet was resuspended in DMEM supplemented with 10% heat-inactivated fetal calf serum (FCS) (PAA Laboratories GmbH, Pasching, Austria) and antibiotics, seeded into culture flasks (Cellstar; Greiner Bio-One GmbH, Frickenhausen, Germany), and incubated in a humidified atmosphere of 5% CO_2 _at 37°C. Culture media were changed twice a week, and preconfluent cells were passaged using accutase (PAA Laboratories GmbH). Early-passage cells (passages 2 to 4) were used for all experiments.

### Immunohistochemical evaluation of the joint capsule biopsies

Specimens of hip joint capsules and contracted elbow joint capsules were fixed in neutral buffered formalin and embedded in paraffin, and 5 μm-sections were routinely stained with hematoxylin-eosin. MFs in the biopsies were detected by immunohistochemical staining for α-SMA using a monoclonal primary mouse anti-α-SMA antibody (dilution 1:600; Progen Biotechnik GmbH, Heidelberg, Germany; clone ASM-1) followed by a ready-to-use biotinylated secondary antibody (Dako Real™ Link; Dako, Glostrup, Denmark) and were visualized using the streptavidin-peroxidase method with 3,3'-diaminobenzidine (DAB) as chromogen. Immunohistochemical staining was performed with an automated staining system (Dako TechMate 500 PLUS; Dako) using a standard ready-to-use kit. Histological slides were evaluated under an Olympus light microscope (BX45; Olympus, Hamburg, Germany) and documented with a digital camera (Camedia C7070; Olympus).

The expression of α-SMA in MF cultures originating from hip joint capsules was verified using a monoclonal mouse anti-human-α-SMA antibody (Dako, Hamburg, Germany). The cells were seeded on histological cover slides at a density of 25,000 cells/cm^2^, incubated for 24 hours, and fixed with 3.7% paraformaldehyde (PFA). The slides were incubated with the primary antibody for 2 hours, washed with PBS, incubated with the secondary horseradish peroxidase-coupled rabbit anti-mouse antibody (Dako), and stained with DAB. Cell nuclei were counterstained with Mayer's hemalun (Merck AG, Darmstadt, Germany). The positive cells have been counted and calculated in subconfluent cultures in five separate viewing fields under a light microscope.

Immunofluorescence analysis of cell culturesMFs (4 × 10^4 ^cells/well) were cultured on histological cover slides with or without the cyclooxygenase-2 (COX2) inhibitor diclofenac (10 μg/mL) in DMEM containing 1% FCS. The expression of α-SMA and COX2 in MFs was determined by immunofluorescence double-staining using a primary monoclonal mouse anti-human-α-SMA antibody (dilution 1:50, clone 1A4; Dako; 45 minutes at room temperature) and a primary mouse anti-human-COX2 antibody (1:100 in saponin buffer, clone 33/COX2; BD Biosciences; 45 minutes at room temperature) in PFA-fixed cell cultures. Cells were washed two times with cold PBS and incubated with Texas Red-conjugated goat anti-mouse IgG2a for α-SMA followed by fluorescein isothiocyanate-conjugated goat anti-mouse IgG1 for COX2 (both from SouthernBiotech, Birmingham, AL, USA) as a secondary antibody. Negative controls were performed using respective isotype antibodies. Cell nuclei were stained with Hoechst 33258 (dilution 1:10,000; Sigma-Aldrich Chemie GmbH) for 10 minutes at room temperature in all experiments. Subsequently, the slides were rinsed and embedded with Gel Mount (SouthernBiotech) on glass cover slides. Cells were visualized using a laser scanning confocal microscope (TCS SP-2; Leica Microsystems, Bensheim, Germany).

### Cell viability and proliferation

**Figure 1:**
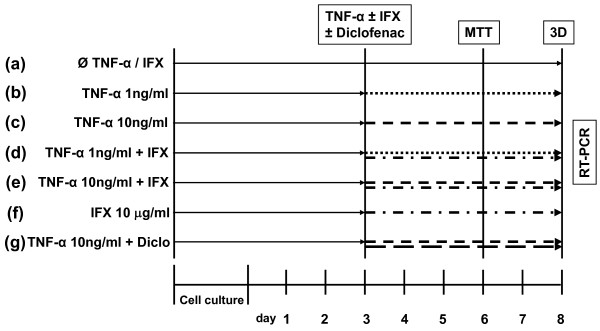
**The experimental setup to study the effect of tumor necrosis factor-alpha (TNF-α) on human joint capsule myofibroblasts**. Seven different groups **(a-g) **were chosen in the study. Group (a) as the control was cultured without any cytokine or inhibitor. The cytokine or the inhibitor or both were added after 3 days of culture. On day 6, the MTT assay was performed and the three-dimensional (3D) collagen gels were released from the culture plate. After 48 hours, gel surfaces were calculated as indicated in Materials and methods. Diclo, diclofenac; IFX, infliximab; MTT, 3-(4,5-dimethylthiazol-2-yl)-2,5-diphenyl tetrazolium bromide; RT-PCR, real-time polymerase chain reaction.

MFs were seeded into 96-well plates (Greiner Bio-One GmbH) at a density of 10 × 10^4 ^cells/well and incubated in 150 μL of serum-supplemented DMEM (5% FCS, 1% penicillin/streptomycin) for 48 hours. Thereafter, the cell layers were washed with PBS and incubated for 24 hours in 150 μL of serum-free DMEM supplemented with 1% bovine serum albumin (BSA). After 24 hours, the cells were washed with PBS and incubated for 72 hours in 150 μL of serum-free DMEM containing 1 or 10 ng/mL of TNF-α (R&D Systems GmbH, Wiesbaden-Nordenstadt, Germany) and/or the chimeric monoclonal antibody to TNF-α (10 μg/mL IFX, generously provided by Centocor B.V., Leiden, The Netherlands), and/or 10 μg/mL of the COX2 inhibitor diclofenac (Figure 1). MFs cultured in 150 μL of serum-free DMEM without any cytokine or inhibitor were used as controls and named as the control group. Cell viability and proliferation were measured using the colorimetric 3-(4,5-dimethylthiazol-2-yl)-2,5-diphenyl tetrazolium bromide (MTT) assay (Promega GmbH, Mannheim, Germany). After 72 hours, 30 μL of 0.5% MTT solution was added to each well and incubated for 2 hours. The medium was removed, and the dye was resolved with 100 μL of isopropanol (Hedinger GmbH & Co. KG, Stuttgart, Germany). The optical density was measured at 570 nm (650 nm background) using an enzyme-linked immunosorbent assay reader (Sunrise; Tecan Deutschland GmbH, Crailsheim, Germany).

### Collagen gel contraction assay

A three-dimensional (3D) collagen gel contraction model, which is a well-accepted method for the estimation of the cell-mediated contracture of the ECM *in vitro *in a 3D environment [21], was established to estimate the contractile forces exhibited by human hip joint MFs. The present protocol was established with primary human hip joint capsule MFs by varying cell numbers, collagen gel volumes and concentrations, time points of detachment, and different concentrations of TGF-β1 as a positive control (data not shown) [18,22,23]. Due to limited numbers of primary cells from each donor, conditions requiring least possible cell numbers and collagen volumes have been used.

Collagen gels were prepared using type I rat collagen (1.5 mg/mL; BD Biosciences, Bedford, MA, USA) in a 10-fold Medium 199 concentrate, 7.5% NaHCO_3_, 1N NaOH (all from Sigma-Aldrich Chemie GmbH), and distilled water. The cells were resuspended in the gel solution and seeded into 24-well plates at a density of 1.2 × 10^5 ^cells/300 μL. After 30 minutes, the solidified gels were incubated in 1 mL of serum-supplemented DMEM (10% FCS) for 48 hours. Thereafter, the gels were washed with PBS and incubated for 24 hours in 1 mL of serum-free DMEM supplemented with 1% BSA. After a vigorous rinsing with PBS, the cells were incubated for 72 hours in 1 mL of serum-free DMEM containing TNF-α and/or IFX and/or diclofenac according to Figure 1. MFs incubated in 150 μL of serum-free DMEM only were used as controls and named as the control group. Thereafter, the gels were released from the culture plate with a pipette tip and cultured for a further 48 hours. Gel surfaces were scanned using the Canon 660 U scanner (Canon Deutschland GmbH, Krefeld, Germany) and calculated using the software ImageJ (National Center for Biotechnology Information, Bethesda, MD, USA).

### Simultaneous quantification of prostanoids using gas chromatography/mass spectrometry

Cells were cultured under the same conditions as described in the previous section (Collagen gel contraction assay) and challenged with TNF-α (1 or 10 ng/mL) as described above or coincubated with TNF-α (10 ng/mL) and diclofenac (10 μg/mL) (Figure 1) after serum deprivation for 24 hours. Cell culture medium supernatants were collected after 24, 48, 72, and 96 hours of culture and fresh medium was subsequently added to the cultures at these time points. The synthesis of prostaglandins E_2 _(PGE_2_), F_1A _(PGF_1A_), and F_2A _(PGF_2A_) was determined by gas chromatography/mass spectrometry (GC/MS). Sample aliquots were kept at -80°C until further analysis. Concentrations of PGE_2_, PGF_2A_, and the stable prostacyclin metabolite 6-keto-PGF_1A _were determined using GC/MS with minor modifications of a previously described method [24]. Briefly, cell culture supernatants were spiked with approximately 10 ng of deuterated internal standards. The methoxime derivatives were obtained by treatment with O-methylhydroxylamine hydrochloride in sodium acetate buffer. After acidification (pH 2.6), analytes were extracted and further derivatized to the correspondent pentafluorobenzyl esters. Samples were purified by thin-layer chromatography, and two broad zones with R_f _0.03 to 0.39 and 0.4 to 0.8 were scraped off and eluted. After withdrawal of the organic solvent, trimethysilyl ethers were prepared by reaction with bis(trimethylsilyl)-trifluoroacetamide and thereafter injected into the GC/MS/MS. We used a Finnigan (Thermo Fisher Scientific GmbH, Dreieich, Germany) MAT TSQ700 GC/MS/MS, equipped with a Varian 3400 gas chromatograph (Varian, Inc., Palo Alto, CA, USA) and a CTC A200s autosampler (CTC Analytics AG, Zwingen, Switzerland). GC of prostanoid derivatives was carried out on a DB-1 (20 m, 0.25 mm ID, 0.25-μm film thickness) capillary column (Analyt GmbH, Mühlheim, Germany) in the splitless injection mode. GC/MS/MS parameters were exactly as described by Schweer and colleagues [24].

### Real-time polymerase chain reaction

Total RNA was extracted and purified from the cells following the 3D collagen gel contraction assay using Trizol (Invitrogen Corporation) and RNeasy Micro Kits (Qiagen GmbH, Hilden, Germany). Reverse transcription was performed using 2 μg of RNA, M-MuLV-reverse transcriptase, and hexamer primers (Peqlab Biotechnologie GmbH, Erlangen, Germany). Real-time polymerase chain reactions (PCRs) were performed using validated QuantiTect^® ^primers (Qiagen GmbH) for *α-SMA *(QT00088102), *collagen type I *(QT00037793), and *18S *RNA (QT00199367), as well as the QuantiTect SYBR^® ^Green quantitative PCR Supermix (Invitrogen Corporation), an ABI 7300 device (Applied Biosystems Deutschland GmbH, Darmstadt, Germany), and the following thermal profile: 15 minutes at 95°C, 40 cycles of 15 seconds at 94°C, 30 seconds at 55°C, and 35 seconds at 72°C, followed by a dissociation step to confirm specificity of the reaction. The results were quantified using the 2^ΔCt ^method and analyzed with the SDS 2.1 software (Applied Biosystems Deutschland GmbH). Measurement values were indicated as fold expression of the housekeeping gene *18S*.

### Western immunoblot

MFs (5 × 10^5 ^cells/medium culture flask) were cultured with or without the COX2 inhibitor diclofenac (10 μg/mL) in DMEM containing 1% FCS. Protein extraction was performed using ice-cold lysis buffer (2 M Tris/HCl, pH 6.8 to 7.5 containing SDS, glycerol, and brome phenol blue). For each sample, 10 μg of protein was denatured, subjected to 10% SDS-polyacrylamide gel electrophoresis, and blotted to a polyvinylidene difluoride membrane (Millipore GmbH, Schwalbach, Germany). The membranes were blocked in Tris/Tween_20 _(TBST pH 7.4) containing 3% milk powder for 1 hour and incubated with primary mouse anti-human antibodies against α-SMA (dilution 1:100, clone 1A4; Dako), COX2 (1:250, clone 33/COX2; BD Biosciences), and β-actin (dilution 1:10,000; Sigma-Aldrich Chemie GmbH) overnight at 4°C. Immunoreactive bands were detected with secondary horseradish peroxidase-conjugated anti-mouse antibodies (diluted 1:5,000; Cell Signaling Technology, Inc./New England Biolabs GmbH, Frankfurt am Main, Germany) and visualized by enhanced chemiluminescence detection reagents (Western Lightning Plus Kit; PerkinElmer Inc., Waltham, MA, USA) on autoradiograph films (Agfa Curix HT 1.000 G Plus; Agfa-Gevaert N.V., Mortsel, Belgium).

### Statistical analysis

All experiments and measurements were performed at least in triplicate, and the number of measurements for each experiment is indicated in the figure legends. For statistical analysis, the SPSS 10.07 software (SPSS Inc., Chicago, IL, USA) was used. The data distribution was defined by medians ± quartiles. For fold comparisons, the measurement values were normalized to the respective individual replicate samples of the control group and transformed to a log2 scale. The data distribution was presented in box plots. For multiple comparisons, the paired non-parametric Wilcoxon test was performed. Differences were considered to be statistically significant for *P *< 0.05 and depicted by *P < 0.05, **P < 0.01, and ***P < 0.001.

## Results

### Contracted elbow joint capsules reveal high numbers of α-SMA-positive cells

**Figure 2:**
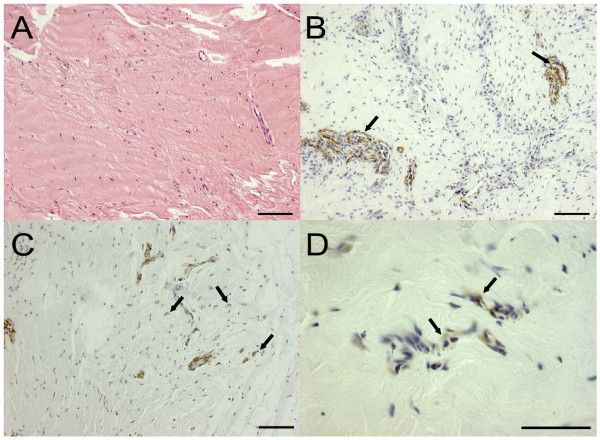
**Expression of the myofibroblast marker alpha-smooth muscle actin (α-SMA) in hip joint and contracted elbow joint capsules**. Biopsy sections were stained as indicated in Materials and methods. **(a) **Hematoxylin-eosin staining of hip joint capsules revealed parallel orientation of the collagen fibers and small spindle-like fibrocytes. **(b) **The immunohistochemical detection of α-SMA in hip joint capsules showed that only smooth muscle cells associated with blood vessels were positive for this marker (arrows). **(c, d) **Arrows indicate multiple positive cells in the immunohistochemical staining for α-SMA (brown dye) in contracted elbow joint capsule which were not linked to blood vessels. Scale bars = 100 μm.

The histological analysis of the biopsies from hip joint capsules yielded a comparable pattern for every patient studied. Beneath the synovial membrane consisting of a monolayer or a multilayer of synoviocytes as well as loose soft tissue with small blood vessels, a thin layer of fat tissue was observed. The layers underneath showed a regularly oriented fiber-rich ECM with low numbers of spindle-like cells that were negative for α-SMA (Figure 2a). The smooth muscle cells of the blood vessels reacted strongly positive with antibodies against α-SMA and thus were used as an internal positive control (Figure 2b). In contrast, the specimens of contracted elbow joint capsules were interspersed with small spindle-like cells that were strongly positive for α-SMA by immunohistochemistry (Figure 2c, d). Each patient studied with contracted capsule revealed comparable α-SMA staining pattern. The soft tissue layer showed a more irregular, partially sclerosing fibrous tissue, occasionally representing mucoid degeneration and lymphocytic infiltrates.

### Mature joint capsule fibroblasts in culture express α-SMA

**Figure 3:**
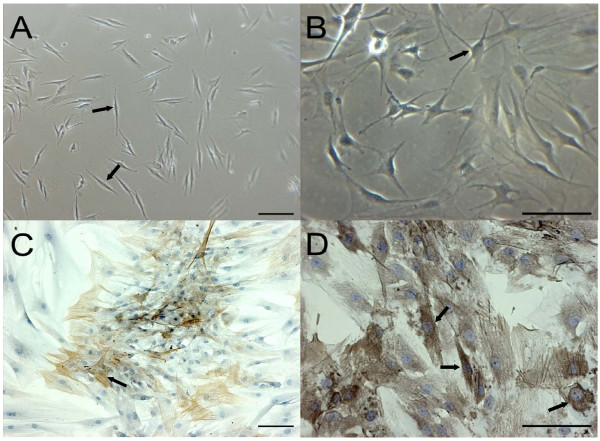
**Phenotype of the cells used in this study**. **(a) **Early cultures of fibroblasts were characterized by a typical spindle-like shape (arrows) and gradually matured into myofibroblasts **(b) **revealing typical stellate-shaped morphology (arrows) over the course of culture. **(c, d) **The myofibroblast cell marker alpha-smooth muscle actin was detected in confluent cell cultures as indicated in Materials and methods. Scale bars = 100 μm.

Only a few days after incubation, the typical spindle-like shape of *in vitro *cultured fibroblasts (Figure 3a) increasingly changed toward the phenotype of stellate cells (Figure 3b), which were strongly positive for the MF marker α-SMA (Figure 3c, d). The positive staining for α-SMA focused on regions of intracellular stress fibers, a hallmark of MFs. Before the start of the experiments, the preconfluent cell cultures contained almost 80% to 100% α-SMA-positive cells (Figure 3d).

### The pro-inflammatory cytokine TNF-α induces myofibroblast proliferation

**Figure 4:**
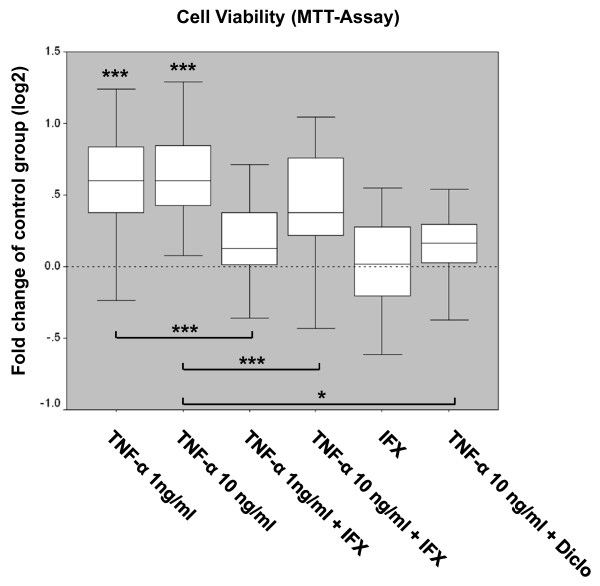
**Cell viability and proliferative capacity of myofibroblasts upon tumor necrosis factor-alpha (TNF-α) treatment**. The effect of the cytokine TNF-α (1 and 10 ng/mL) in the presence or absence of the TNF-α inhibitor infliximab (IFX) (10 μg/mL) or the cyclooxygenase inhibitor diclofenac (Diclo) on myofibroblasts was analyzed by using the MTT cell viability assay. Data are representative of nine (Table 1) independently performed TNF-α ± IFX experiments and seven independently performed TNF-α ± diclofenac experiments with four replicate measurements from each individual patient sample (n = 68 for the 'control' and 'TNF-α 10 ng/mL' groups, n = 28 for the 'TNF-α 10 ng/mL+diclofenac' group, and n = 36 for all other groups). Results are plotted as fold changes of the respective samples in the control group according to the paired non-parametric Wilcoxon test used for the statistical analysis. *P < 0.05, ***P < 0.001. MTT, 3-(4,5-dimethylthiazol-2-yl)-2,5-diphenyl tetrazolium bromide.

Upon addition of TNF-α, MF cultures revealed a dose-dependent increase of cell viability and proliferation (Figure 4). Compared with the control group, cell proliferation was significantly induced by 1 ng/mL TNF-α and did not notably increase proliferation at the higher concentration of the cytokine (Figure 4 and Table 1). The proliferative effect of TNF-α (1 or 10 ng/mL) was significantly reduced by its blocker IFX (10 μg/mL). MFs cultured with IFX only showed no significant differences in terms of cell viability compared with the control group (Figure 4 and Table 1). Interestingly, coincubation of MFs with TNF-α (10 ng/mL) and the COX2 inhibitor diclofenac (10 μg/mL) resulted in a significant inhibition of TNF-α-induced cell proliferation.

**Table 1:** Data distribution and statistical analysis

			Fold change of the respective patient sample in the control group (log2)			
				
Experiment	Group	Number^a^	25th quartile	Median	75th quartile	*P *value
MTT assay	B: 1 ng/mL TNF-α	36	0.37	0.60	0.85	B vs. A: <0.001
	C: 10 ng/mL TNF-α	68	0.43	0.60	0.85	C vs. A: <0.001
	D: 1 ng/mL TNF-α + 10 μg/mL IFX	36	-0.03	0.13	0.39	D vs. B: <0.001
	E: 10 ng/mL TNF-α + 10 μg/mL IFX	36	0.21	0.38	0.78	E vs. C: <0.001
	F: 10 μg/mL IFX	36	-0.21	0.02	0.30	F vs. A: 0.2
	G: 10 ng/mL TNF-α + 10 μg/mL Diclo	28	0.02	0.16	0.30	G vs. C: 0.031
						
3D collagen gel	B: 1 ng/mL TNF-α	40	-0.01	0.23	0.46	B vs. A: <0.001
	C: 10 ng/mL TNF-α	52	0.71	0.90	1.47	C vs. A: <0.001
	D: 1 ng/mL TNF-α + 10 μg/mL IFX	40	-0.44	-0.22	0.04	D vs. B: <0.001
	E: 10 ng/mL TNF-α + 10 μg/mL IFX	40	-0.35	-0.14	0.19	E vs. C: <0.001
	F: 10 μg/mL IFX	40	-0.23	-0.01	0.15	F vs. A: 0.6
	G: 10 ng/mL TNF-α + 10 μg/mL Diclo	40	0.04	0.17	0.67	G vs. C: 0.002
						
qPCR						

*α-SMA*	B: 1 ng/mL TNF-α	8	-1.01	-0.14	1.15	B vs. A: 0.8
	C: 10 ng/mL TNF-α	25	-6.29	-5.64	-3.48	C vs. A: <0.001
	D: 1 ng/mL TNF-α + 10 μg/mL IFX	8	-1.20	-0.004	0.90	D vs. B: 0.4
	E: 10 ng/mL TNF-α + 10 μg/mL IFX	8	-0.63	-0.50	0.10	E vs. C: 0.01
	F: 10 μg/mL IFX	8	-0.45	-0.32	-0.12	F vs. A: 0.5
	G: 10 ng/mL TNF-α + 10 μg/mL Diclo	9	-4.85	-4.32	-3.74	G vs. C: 0.015
						
*Collagen type I*	B: 1 ng/mL TNF-α	8	-0.36	-0.007	0.59	B vs. A: 1.0
	C: 10 ng/mL TNF-α	17	-4.35	-3.47	-3.00	C vs. A: 0.01
	D: 1 ng/mL TNF-α + 10 μg/mL IFX	8	-0.93	-0.30	0.54	D vs. B: 0.2
	E: 10 ng/mL TNF-α + 10 μg/mL IFX	8	-1.03	-0.20	0.50	E vs. C: 0.01
	F: 10 μg/mL IFX	8	-1.60	-0.009	0.27	F vs. A: 0.6
	G: 10 ng/mL TNF-α + 10 μg/mL Diclo	9	-2.21	-1.84	-0.93	G vs. C: 0.015

^a^Number of measurements. 3D, three-dimensional; α-SMA, alpha-smooth muscle actin; Diclo, Diclofenac; IFX, infliximab; MTT, 3-(4,5-dimethylthiazol-2-yl)-2,5-diphenyl tetrazolium bromide; qPCR, quantitative polymerase chain reaction; TNF-α, tumor necrosis factor-alpha.

### TNF-α inhibits contractile forces exhibited by myofibroblasts

**Figure 5:**
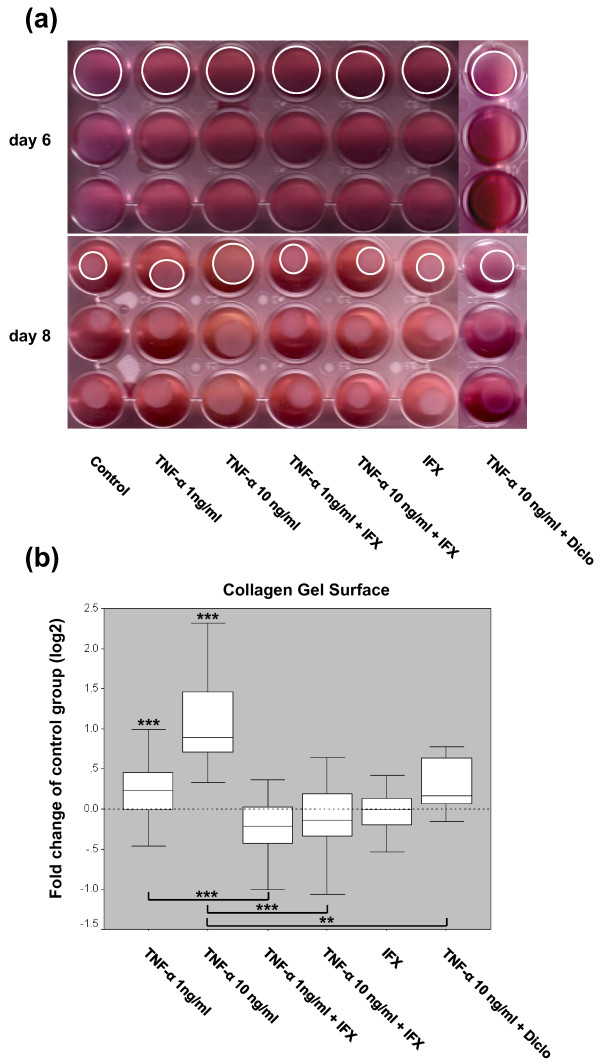
**The effect of tumor necrosis factor-alpha (TNF-α) on collagen gel contraction**. **(a) **Images of the three-dimensional collagen gel contraction assay shown are representative of all (Table 1) independently performed experiments. Three collagen gels of each group are shown before (day 6) and after (day 8) releasing the gels from the culture plates, revealing distinct differences in gel contraction in the groups upon TNF-α and infliximab (IFX) or diclofenac (Diclo) coincubation. White circles in the first rows of the plates illustrate the margins of the gels comprising the gel surface areas. **(b) **Gel surfaces of the floating gels in the presence or absence of TNF-α (1 and 10 ng/mL), the TNF-α inhibitor IFX (10 μg/mL), or diclofenac (10 μg/mL) were scanned and calculated as described in Materials and methods. Data are representative of 10 (Table 1) independently performed TNF-α ± IFX experiments and three independently performed TNF-α ± diclofenac experiments with four replicate measurements from each individual patient sample (n = 52 for the 'control' and 'TNF-α 10 ng/mL' groups, n = 12 for the 'TNF-α 10 ng/mL+diclofenac' group, and n = 40 for all other groups). Results are plotted as fold changes of the respective samples in the control group according to the paired non-parametric Wilcoxon test used for the statistical analysis. **P < 0.01, ***P < 0.001.

In comparison with the controls, the addition of 1 ng/mL TNF-α significantly inhibited collagen gel contraction as the gel surfaces of this group were significantly larger (Figure 5a, b and Table 1). This effect is indicative of reduced contractile forces exhibited by MFs. The inhibition of collagen gel contracture was even stronger upon the application of 10 ng/mL TNF-α. This inhibitory effect of the cytokine was significantly blocked by IFX as the surface areas of the collagen gels reversed to a dimension that was comparable to the controls. MFs cultured with IFX only showed no significant change of contraction behavior compared with the controls (Figure 5a, b and Table 1). The addition of diclofenac to collagen gels that were stimulated with 10 ng/mL TNF-α before significantly reversed the TNF-α effect and promoted ECM contraction (Figure 5a, b and Table 1).

### TNF-α suppresses α-SMA and collagen type I gene expression in myofibroblasts

**Figure 6:**
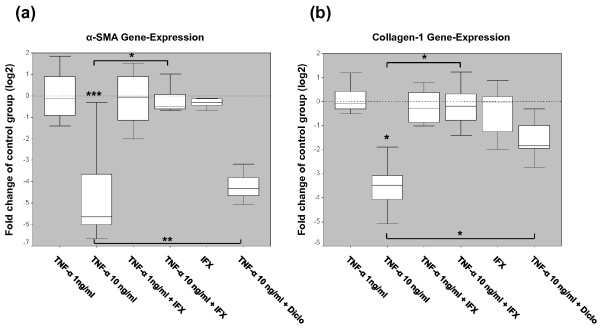
**Tumor necrosis factor-alpha (TNF-α) downregulates gene expression of the myofibroblast marker *alpha-smooth muscle actin (α-SMA) *and the extracellular matrix protein *collagen type I***. The mRNA expression of *α-SMA ***(a) **and *collagen type I ***(b) **was determined for every group (Figure 1) by quantitative real-time polymerase chain reaction. The mRNA level of *α-SMA *of every group examined was normalized to the housekeeping gene *18S*. Data are representative of four (Table 1) independently performed TNF-α ± infliximab (IFX) experiments and TNF-α ± diclofenac (Diclo) experiments with duplicate measurements from each individual patient sample (n = 16 for the 'control' and 'TNF-α 10 ng/mL' groups and n = 8 for all other groups). Results are plotted as fold changes of the respective samples in the control group according to the paired non-parametric Wilcoxon test used for the statistical analysis. *P < 0.05, **P < 0.01, ***P < 0.001.

Whereas the lower concentration of TNF-α revealed low inhibitory effects on gene expression of *α-SMA *and *collagen type I*, the expression of these two transcripts was significantly downregulated upon addition of 10 ng/mL TNF-α (Figure 6a, b and Table 1). This suppressive effect on gene expression of these two markers was blocked by IFX, as the expression of both genes reverted almost to the level of the control group, and was significantly reduced by the COX2 inhibitor diclofenac. However, IFX alone did not influence the gene expression of *α-SMA *and *collagen type I *(Figure 6a, b and Table 1).

### The effects of TNF-α on myofibroblasts are mediated by prostaglandin E2 synthesis

**Figure 7:**
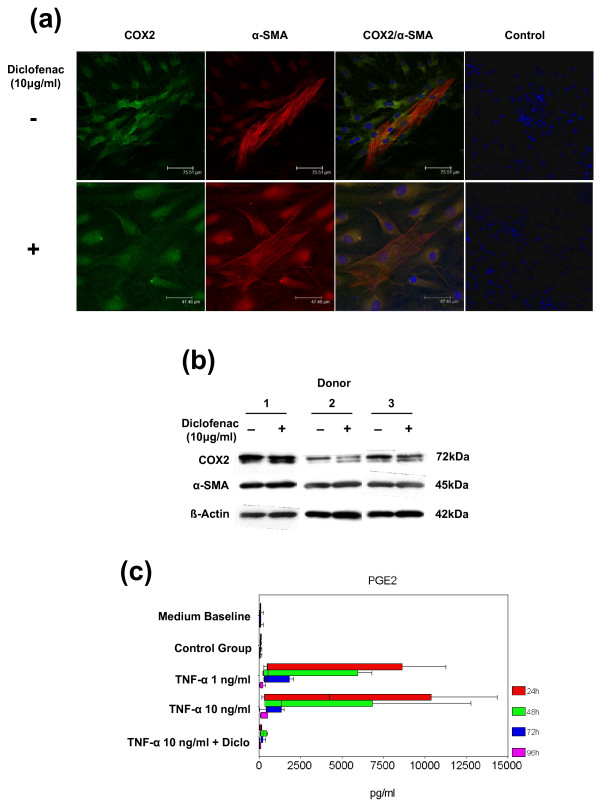
**Tumor necrosis factor-alpha (TNF-α) modulates the synthesis of prostaglandin E_2 _(PGE_2_) in alpha-smooth muscle actin (α-SMA)-positive myofibroblasts (MFs)**. **(a) **Immunofluorescent staining of cyclooxygenase-2 (COX2) (first panel, green, fluorescein isothiocyanate filter) and α-SMA (second panel, red, Texas Red filter) in MF cultures with or without the COX2 inhibitor diclofenac (Diclo). The third panel illustrates merged images of Höchst 33248-stained nuclei (blue, DAPI filter) as well as immunofluorescence for COX2 and α-SMA. α-SMA-positive MFs derived from hip joint capsules express the enzyme COX2. The fourth panel shows merged images of the negative controls. Scale bars are shown in the lower right corner of each panel. **(b) **MFs of three different donors were exposed to 10 μg/mL diclofenac. The expression of α-SMA (45 kDa), COX2 (72 kDa), and β-actin (42 kDa) as a loading control was evaluated by using Western blots. A characteristic double band for the COX2 protein corresponding to the expected molecular weight represents different glycosylated forms of the enzyme. **(c) **Gas chromatographic/mass spectrometric analysis revealed a time-dependent increase of PGE_2 _concentration in MF cultures upon TNF-α stimulation with a peak after 24 and 48 hours. This effect was completely blocked by diclofenac. DAPI, 4'-6-diamidino-2-phenylindole.

Using immunfluorescence staining and Western blot analysis, we found that α-SMA-positive human MFs did express the enzyme COX2 (Figure 7a, b), which is required for the synthesis of PGE_2_. GC/MS analysis revealed a dramatic time-dependent increase of PGE_2 _concentrations in MF cultures upon stimulation with TNF-α. Low and high concentrations of TNF-α yielded a comparable synthesis level of PGE_2_, with a peak response after 24 and 48 hours (Figure 7c). Interestingly, the syntheses of both PGF_1A _and PGF_2A _(data not shown) were not affected by TNF-α. The PGE_2 _levels in the control group were as low as in culture medium without cells, indicating that only low levels of PGE_2 _were synthesized during basal culture conditions. Coincubation of MFs with TNF-α (10 ng/mL) and diclofenac (10 μg/mL) resulted in a complete abrogation of the TNF-α-mediated increase in PGE_2 _synthesis (Figure 7c), which was associated with a significant decline in TNF-α-induced effects on cell proliferation (Figure 4), ECM contraction (Figure 5b), and *collagen type I *gene expression (Figure 6b). Although the TNF-α-mediated inhibition of *α-SMA *gene expression was also attenuated by coincubation with diclofenac, the treatment with diclofenac did not reduce the overall effects of TNF-α on MF cell function to the same extent as IFX (Figure 6a, b). Furthermore, treatment of MFs with 10 μg/mL diclofenac only did not reveal any significant effects on the protein expression of COX2 or α-SMA (Figure 7a, b).

## Discussion

Tissue healing is a complex process that requires activation, migration, and differentiation of various cells that are capable of ECM synthesis and later wound repair. Therefore, transformation of fibroblasts to contractile MFs is generally accepted to be a key element in early wound healing. In this study, we could show that in contrast to hip joint capsules of patients who did not suffer from any condition known to affect the ROM of the respective joints, the number of α-SMA-positive MFs is notably increased in the biopsies of contracted joint capsules after injury. To therapeutically counteract an excessive ECM synthesis and contraction, it is most important to understand the molecular pathways that regulate the activation and function of MFs.

Over the years, it has become evident that MFs arise from a variety of sources and may develop different phenotypes according to the involved organ and the physiological or pathological situation [9,18,25,26]. With respect to the current literature, the origin of the cells as well as their cytokine environment [9] are decisive for understanding MF development and regulation. Although TNF-α was shown to mediate different target cells in the pathogenesis of fibrocontractive disorders, the role of this cytokine in the pathogenesis of post-traumatic joint contracture has not been defined yet.

Here, we describe in detail the functional effect of TNF-α on human MFs that differentiated from fibroblasts isolated from hip joint capsules. Although it has been previously described that normal elbow capsules can be obtained from organ donors [7] for limited resources, we did not take capsules of organ donors as a source of MFs for our functional experiments. We could demonstrate that TNF-α is capable of inducing cell viability and proliferation in MF cultures. This effect was already present at a low concentration of the cytokine. However, the stimulation of the cells with higher concentrations of TNF-α did not result in additional increase of the cell proliferation rate, presumably due to complete receptor saturation. Furthermore, as the proliferative effect of TNF-α was significantly reduced by its inhibitor IFX, we conclude that cell proliferation was specifically mediated by this cytokine. On the other hand, we did not observe any significant effects of IFX without TNF-α on cell viability and proliferation in MF cultures. Although our results are consistent with previous findings that TNF-α has the potential to induce proliferation of fibroblasts and MFs [27,28], there is also significant evidence of the antiproliferative effect of TNF-α as previously described in liver MFs, the hepatic stellate cells (HSCs) [29]. Such differences in regulation processes emphasize once more the concept of tissue-specific regulation of MF function.

Despite this positive stimulatory effect on cell proliferation, we found that the contractile forces of MFs were significantly inhibited upon application of TNF-α according to a significant inhibition of *α-SMA *gene expression. This fact supports the hypothesis that the fibroblast-to-MF transition may be affected by TNF-α. Studies in the past revealed that the contractile function of the MFs is linked to the expression of α-SMA and different ECM proteins like collagen type I. According to a previous study on rat lung fibroblasts [30], we found that the functional inhibition of ECM contraction by human joint capsule MFs upon TNF-α treatment was clearly associated with a significant downregulation of *α-SMA *and *collagen type I *gene expression. Interestingly, whereas the lower concentration of TNF-α induced proliferation and significant inhibition of contractile forces in MFs in the collagen gels, we did not find a significant inhibition of *α-SMA *and *collagen I *gene expression at the lower level of this cytokine. Previous studies provided evidence that the decrease in collagen synthesis occurred without a change in cell number [20], indicating that the inhibition of the ECM contraction might be due to decreased synthesis at the cellular level. The synthesis of collagen is a hallmark of the strict regulation of MFs by a complex cytokine environment. Different studies revealed that the pro-inflammatory cytokines TNF-α and IL-1β have profound effects on collagen metabolism in fibroblasts *in vitro *by downregulating the synthesis of collagen [18,20,31]. Singer and Clark [32] demonstrated that TNF-α was able to modulate ECM turnover by inhibition of protein synthesis and activation of matrix metalloproteinases. Moreover, there is evidence that TNF-α is capable of antagonizing TGF-β1-induced upregulation of type I and III collagen expression in mouse fibroblasts [33]. Thus, the antagonistic relationship between TGF-β1 and TNF-α may play an important role in maintaining tissue homeostasis and ECM deposition, whereas the cytokine network that modulates this process is presumably more complex. Based on these data, we believe that the inhibition of ECM contraction by TNF-α might be due to a functional inhibition of human MFs both by a reduced expression level of *α-SMA *as well as by the decrease of ECM protein expression.

The antiproliferative impact of TNF-α in human HSCs, the MFs of the liver, was previously described to be mediated by increased formation of nuclear factor-kappa-B (NF-κB) DNA-binding complexes [29]. Interestingly, the promoter region of the *COX2 *gene possesses specific binding sites for the NF-κB complexes. Thus, treatment of HSCs with TNF-α was shown to positively regulate gene expression of *COX2 *and this accounted for basal COX activity in these cells [29]. On the other hand, exogenous treatment of fetal and adult lung fibroblast with PGE_2 _was reported to inhibit TGF-β1-induced expression of α-SMA and collagen type I by increasing cAMP production [34]. Based on these findings, we hypothesized that the effects of TNF-α on human joint capsule MFs may be mediated even by endogenous prostaglandin synthesis. Here, we could clearly show that human joint capsule MFs express COX2 under *in vitro *conditions (Figure 7a, b) and significantly upregulate synthesis of PGE_2 _(Figure 7c), but not PGF_1A _and PGF_2A_, upon TNF-α treatment. The TNF-α-mediated increase of PGE_2 _levels was completely blocked by the non-specific COX inhibitor diclofenac administered in a concentration that is comparable to therapeutic but not toxic levels in humans [35]. Although some functional parameters of MFs were nearly completely restored by coincubation of TNF-α-treated cells with diclofenac (Figure 7c), the gene expressions of *α-SMA *and *collagen type I *(Figure 6a, b) were still significantly inhibited, suggesting that the effect of TNF-α could not be blocked at this concentration to the same extent as by the neutralizing antibody IFX.

## Conclusions

**Figure 8:**
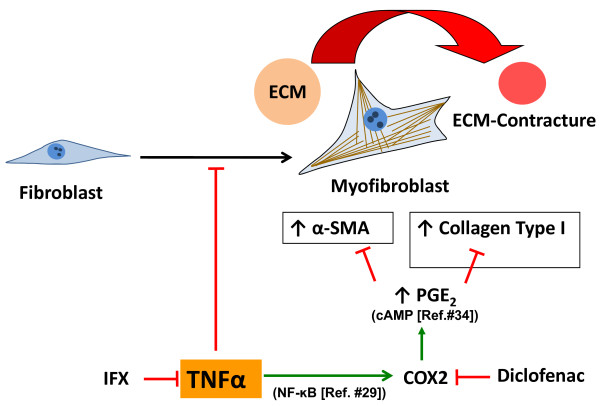
**Illustrative concept of myofibroblast (MF) modulation by tumor necrosis factor-alpha (TNF-α)**. TNF-α inhibits extracellular matrix (ECM) contraction by the downregulation of *alpha-smooth muscle actin (α-SMA) *and *collagen type I *expression in MFs presumably by promoting prostaglandin E_2 _(PGE_2_) synthesis. Both infliximab (IFX) by blocking TNF-α and diclofenac by inhibiting cyclooxygenase-2 (COX2) might enhance ECM contraction. NF-κB, nuclear factor-kappa-B.

In the present work, TNF-α modified the function of human MFs, suggesting a regulative effect of TNF-α during the wound healing. Whereas TNF-α had a high proliferative effect on MFs at low concentrations already, it functionally inhibited the contraction of the ECM and downregulated the gene expression of *α-SMA *and *collagen type I*. As these two genes were only significantly inhibited upon high concentrations of TNF-α stimulation compared with the control group, we conclude that TNF-α might have a dual, dose-dependent modulatory effect on MFs at the beginning of a healing process. The effects of TNF-α on human joint capsule MFs (positive for the marker COX2) were associated with a significant increase of PGE_2 _synthesis, suggesting its crucial role in the regulation of this cell type. Accordingly, they were almost completely prevented by the inhibition of COX2 with diclofenac at a clinically relevant concentration. Our current concept of MF modulation is summarized and illustrated in Figure 8. Further evaluation is required in order to address the hypothesis that cell proliferation and MF function may be altered in clinical use by therapeutically applied immunomodulatory treatments. This established culture and 3D model potentially provide a basis for further investigations to shed new light on this complex cytokine network and to develop alternative non-operative treatment strategies of fibroconnective pathologies like post-traumatic contracture. Prophylactic pharmacological intervention could provide new specific treatment options for post-traumatic contractures and other fibroconnective pathologies.

## Abbreviations

3D: three-dimensional; α-SMA: alpha-smooth muscle actin; BSA: bovine serum albumin; COX2: cyclooxygenase-2; DAB: 3,3'-diaminobenzidine; DMEM: Dulbecco's modified Eagle's medium; ECM: extracellular matrix; FCS: fetal calf serum; GC/MS/MS: gas chromatography/mass spectrometry/mass spectrometry; HSC: hepatic stellate cell; IFX: infliximab; MF: myofibroblast; MTT: 3-(4,5-dimethylthiazol-2-yl)-2,5-diphenyl tetrazolium bromide; NF-κB: nuclear factor-kappa-B; PBS: phosphate-buffered saline; PCR: polymerase chain reaction; PFA: 3.7% paraformaldehyde; PGE_2_: prostaglandin E_2_; PGF: prostaglandin F; ROM: range of motion; TGF-β1: transforming growth factor-beta 1; TNF-α: tumor necrosis factor-alpha.

## Competing interests

The authors declare that they have no competing interests.

## Authors' contributions

SGM helped to design the study and to prepare the final manuscript, carried out all of the experiments with myofibroblasts, and is the project leader. AH helped to design the study and to prepare the final manuscript, contributed to the establishment of the MTT assay and the 3D collagen gel model, and helped to perform the statistical analysis and the interpretation of the results. JW contributed to the establishment of the MTT assay and the 3D collagen gel model and helped to perform the statistical analysis and the interpretation of the results, to generate the hypothesis regarding the role of COX2 and PGE_2 _synthesis, and to perform the respective experiments. SK assisted in cell isolation and cell culture and helped to perform the MTT assays. UR participated in the design of the study, carried out the real-time PCR experiments, and assisted in performing the immunofluorescence analysis. CB carried out the immunohistochemistry, participated in writing the manuscript, and helped to generate the hypothesis regarding the role of COX2 and PGE_2 _synthesis and to perform the respective experiments. BW helped to generate the hypothesis regarding the role of COX2 and PGE_2 _synthesis and to perform the respective experiments. HS-K provided a vial of infliximab and participated in writing the manuscript. LPM participated in writing the manuscript and helped to coordinate the study and to collect capsule biopsies during the surgeries. PMR participated in writing the manuscript, helped to coordinate the study and to collect capsule biopsies during the surgeries, and is the principal investigator. All authors read and approved the final manuscript.
